# Supplementary material for: Silk fibroin hydrogel adhesive enables sealed-tight reconstruction of meniscus tears
Source: Nat Commun. 2024 Mar 26;15:2651. doi: 10.1038/s41467-024-47029-6 (PMC10966011; doi:10.1038/s41467-024-47029-6)
Supplement: Supplementary file 1 — Supplementary Information [file 41467_2024_47029_MOESM1_ESM.pdf]

Supplementary Information

Silk Fibroin Hydrogel Adhesive Enables Sealed-tight Reconstruction of Meniscus Tears

Xihao Pan[1,2,3,4,#], Rui Li[1,3,#], Wenyue Li[1,2,3,#], Wei Sun[1,2], Yiyang Yan[1,2,3], Xiaochen Xiang[3], Jinghua Fang[5], Youguo Liao[1,2], Chang Xie[1,2], Xiaozhao Wang[1,2,3,4], Youzhi Cai[6], Xudong Yao[7], Hongwei Ouyang[1,2,3,4]*

Affiliations:

1. Department of Sports Medicine of the Second Affiliated Hospital, and Liangzhu Laboratory, Zhejiang University School of Medicine, Hangzhou, China.

2. Dr. Li Dak Sum & Yip Yio Chin Center for Stem Cells and Regenerative Medicine, Zhejiang University School of Medicine, Hangzhou, China.

3. Zhejiang University-University of Edinburgh Institute, Zhejiang University School of Medicine, Haining, China.

4. China Orthopedic Regenerative Medicine Group (CORMed), Hangzhou, China.

5. Orthopedics Research Institute, Zhejiang University, Hangzhou, 310009, China.

6. Sports Medical Center, the First Affiliated Hospital, School of Medicine, Zhejiang University, Hangzhou, 310009, China

7. The Fourth Affiliated Hospital, International Institutes of Medicine, Zhejiang University School of Medicine, Yiwu 322000, Zhejiang, China

# These authors contribute equally.

* Correspondence: hwoy@zju.edu.cn (Hongwei Ouyang)

This file includes:

Materials and methods, and Supplementary Figures 1-20, and Tables 1-2 and Movies 1-2.

Materials and methods

Materials

4-(bromomethyl)phenylboronic acid and 1-vinyl imidazole were purchased from Sigma-Aldrich. Raw silk was provided by Zhejiang Xingyue Biotechnology CO., LTD. Glycidyl methacrylate and deuteroxide were purchased from Sigma-Aldrich. Lithium bromide was obtained from Macklin. Calcein-AM/PI double staining kit and Cell Counting Kit-8 (CCK-8) were purchased from *Dojindo and* Beyotime, respectively. Phosphate buffer solution and Trizol reagent were obtained from Cellmax and Invitrogen, respectively. Antibodies used For immunofluorescence staining, cryo-sectioned samples were incubated with Anti-Collagen ll antibody (diluted 1:100, NB600-844, NOVUS, USA) and Anti-Collagen l antibody (diluted 1:200, ab88147, Abcam, USA). Secondary antibodies used are as follows: Goat anti-mouse IgG H&L (Alexa Fluor® 555, ab150114, Abcam, USA) and Goat Anti-Mouse IgG H&L (Alexa Fluor® 488, ab150113 Abcam, USA).

Cell experiments

L929 cell lines were purchased from iCell Bioscience (Shanghai, China) and derived from mice. L929 cell lines were authenticated by the supplied using Short Tandem Repeat test. The rabbit meniscus cells were extracted from the 16-week-old New Zealand white rabbit and cultured with F12 medium. The cell line sources did not take gender into account.

Each 150 μL precursor solution formed hydrogel into each pore of a 24-well plate. The 10000 rabbit meniscus cells or L929 fibroblasts were cultured in immersed medium or on the hydrogels. The cells were observed through the live/dead staining on Day 1, Day 3 and Day 7. Live death assay and immunofluorescence images was recored by inverted microscope (Nikon-LV150N, Nikon, Japan). The 5000 rabbit meniscus cells were cultured in each pore of 96-well plates. The CCK-8 assay was performed to determine the cell viability at Day 1, Day 3, and Day 7. 50,000 rabbit meniscus cells were treated with 8 kinds of growth factors, including BMP7, PDGF-AB, IGF-1, BMP2, BMP2, TGF-β1, bFGF, CTGF and TGF-β3. After 3 days, immunofluorescence was used to verify the secretion of

collagen 1 and collagen 2. About 40,000 rabbit meniscus cells were encapsulated in 200 μL S-Gel and S-PIL10+GF groups. After the culture of 14d, real-time polymerase chain reaction (PCR) was performed to detect the meniscus-related expression *(Sox9*, *Col2a1* and *ACAN*). After the culture of 14d, real-time quantitative polymerase chain reaction (PCR) was performed to detect the meniscus-related expression, including type 2 Collagen (*COL2A1*), SRY (sex-determining region Y)-box 9 (*SOX9*), Agrecan(*ACAN*). All the primers were purchased from Beijing Tsingke Biotech Co., Ltd. used as follow:

COL2A1, Forward: 5′GTC TGT GAC ACT GGG ACT GT 3′ and Reverse: 5′TCT CCG AAG GGG ATC TCA GG 3′

SOX9,Forward: 5'GGC GGA GGA AGT CGG TGA AGA A 3' and Reverse: 5′ GCT CAT GCC GGA GGA GGA GTG T 3′

ACAN, Forward: 5′ CTG CAG ACC AGG AGG TAT GTG A 3′ and Reverse: 5′ GTT GGG GCG CCA GTT CTC AAA T 3′

Controlled drug release

500 ng TGF-β1 was dissolved in 1 mL S-Gel or S-PIL10 precursor solution. Every 160 μl of solution formed hydrogel, which was then soaked in 4 mL of PBS. The 0.5 ml of liquid was taken out at each time point to determine the concentration of growth factor in the solution and meanwhile, 0.5 ml of PBS was added up to 4 mL.

Intracellular antioxidant ability of hydrogels

To investigate the intracellular oxidative stress protection of the hydrogels, we used a ROS probe DCFH-DA to assess intracellular ROS levels. Typically, L929 fibroblasts were seeded at a density of 1.5×10^4 cells per well in confocal dishes and incubated for 12 hours. The plates were then reinjected with a fresh medium containing the same volume of either H_2O_2 or H_2O_2 with hydrogel treatment. Next, cells were stained with DCFH-DA for 30 minutes according to the manufacturer's instructions. Finally, a confocal laser scanning microscope (A1, Nikon, Japan) was used to observe DCFH-DA fluorescence in the cells.

Supplementary Figures and Tables

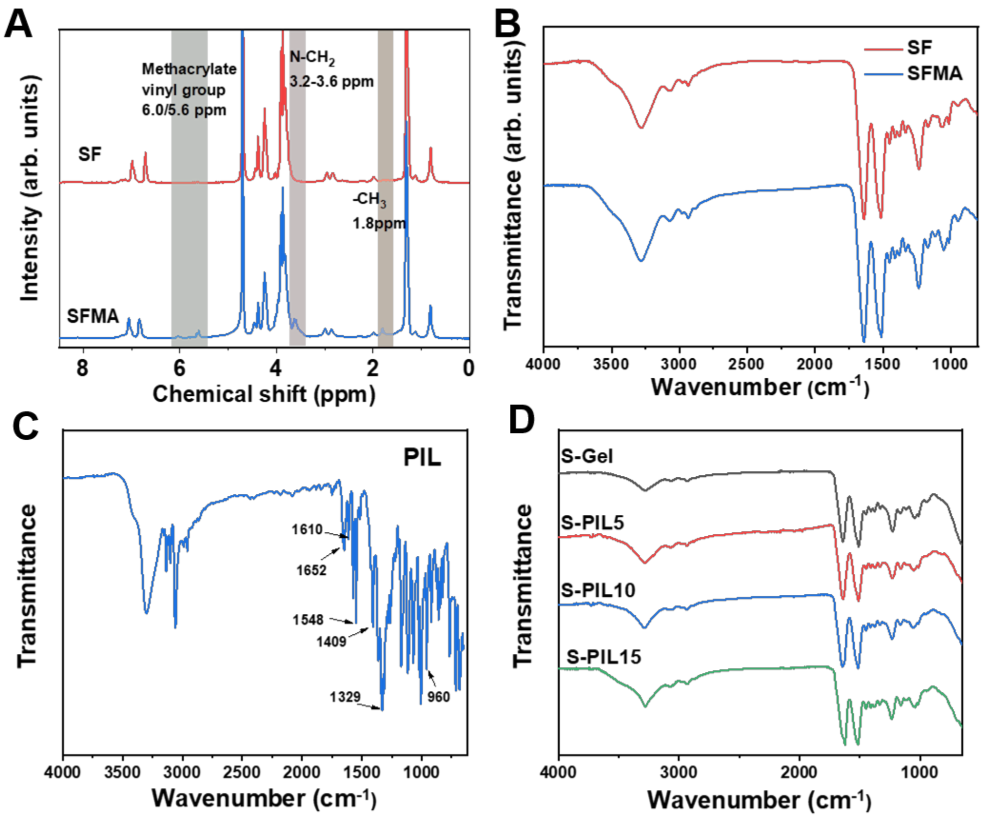

Supplementary Fig. 1 (A) ^{1}H-NMR spectra of silk fibroin (SF) and methacrylated silk fibroin (SFMA). (B) FTIR spectrum of SF and SFMA. (C) FTIR spectrum of PIL. (D) FTIR spectrum of S-Gel and S-PIL gels.

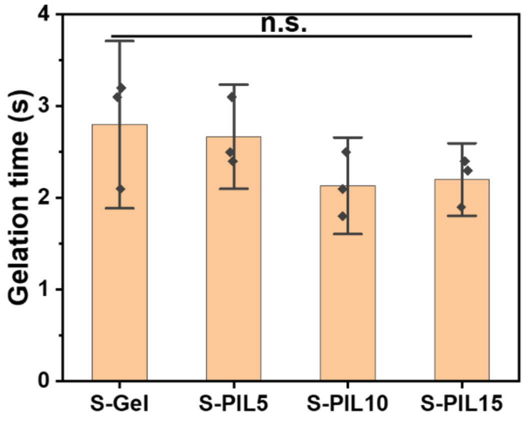

Supplementary Fig. 2 The gelatin time of S-Gel and S-PIL gels. Data are presented as mean ± SD (n = 3 independent experiments). Statistically significant differences, as analyzed using ANOVA followed by Tukey's multiple comparison test, have been indicated as ns: no significance.

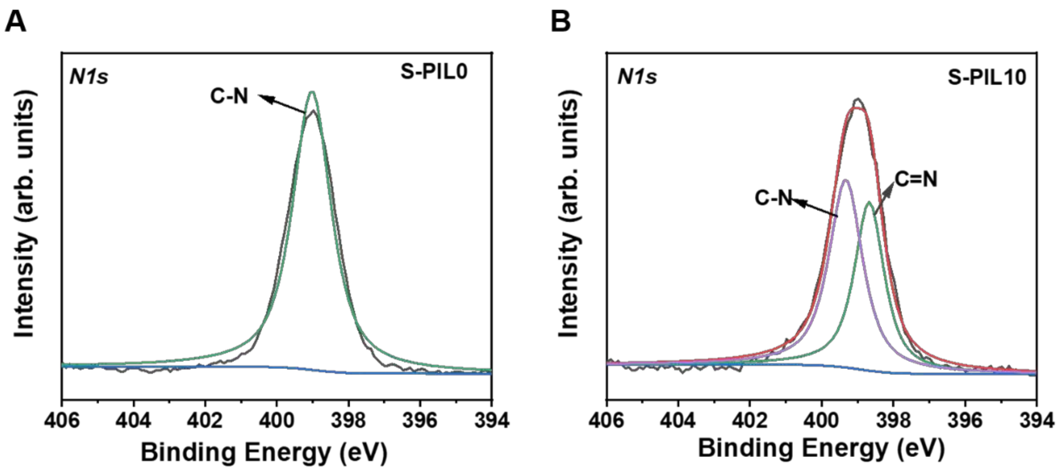

Supplementary Fig. 3 *N1s* XPS signal of (A) S-Gel and (B) S-PIL10.

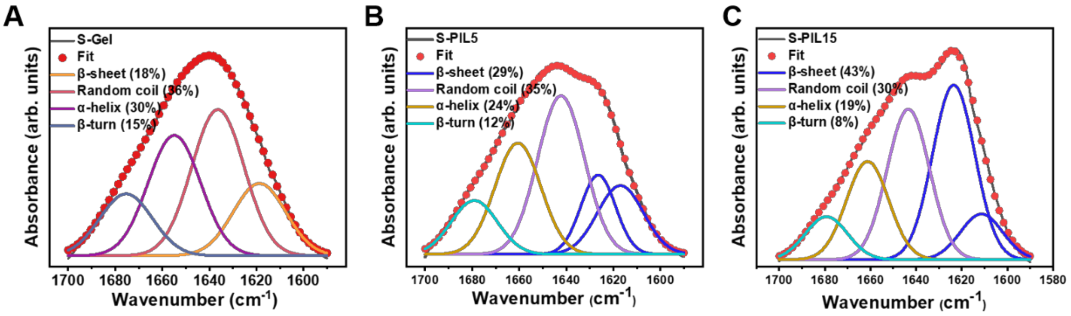

Supplementary Fig. 4 Quantitative analysis of secondary structures of (A) S-Gel, (B) S-PIL5 and (C) S-PIL15.

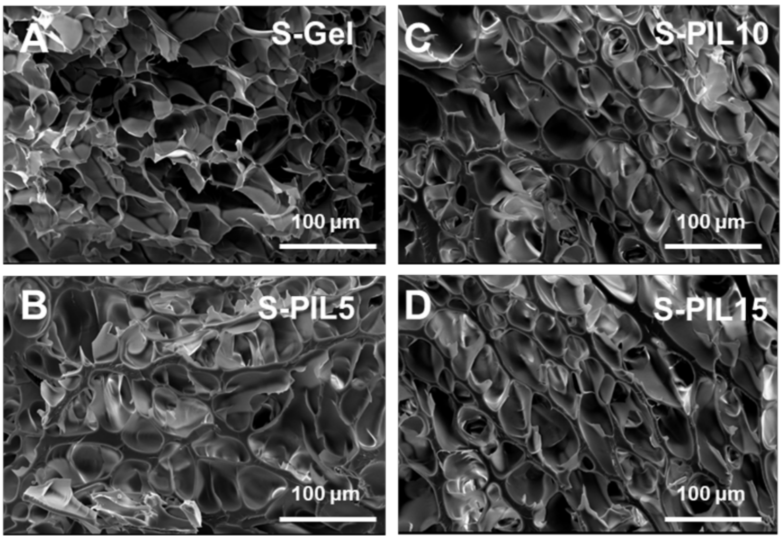

Supplementary Fig. 5 SEM images of (A) S-Gel, (B) S-PIL5, (C) S-PIL10 and (D) S-PIL15.

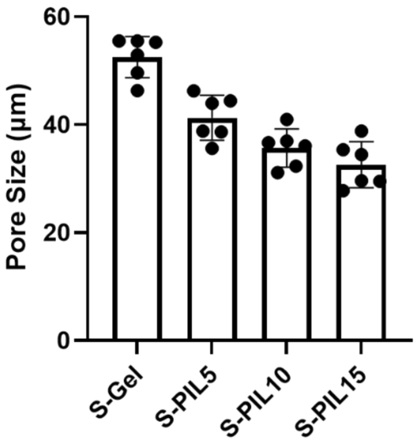

Supplementary Fig. 6 Pore size of S-Gel and S-PIL gels in SEM images. Data have been presented as mean ± SD, n = 6 independent experiments.

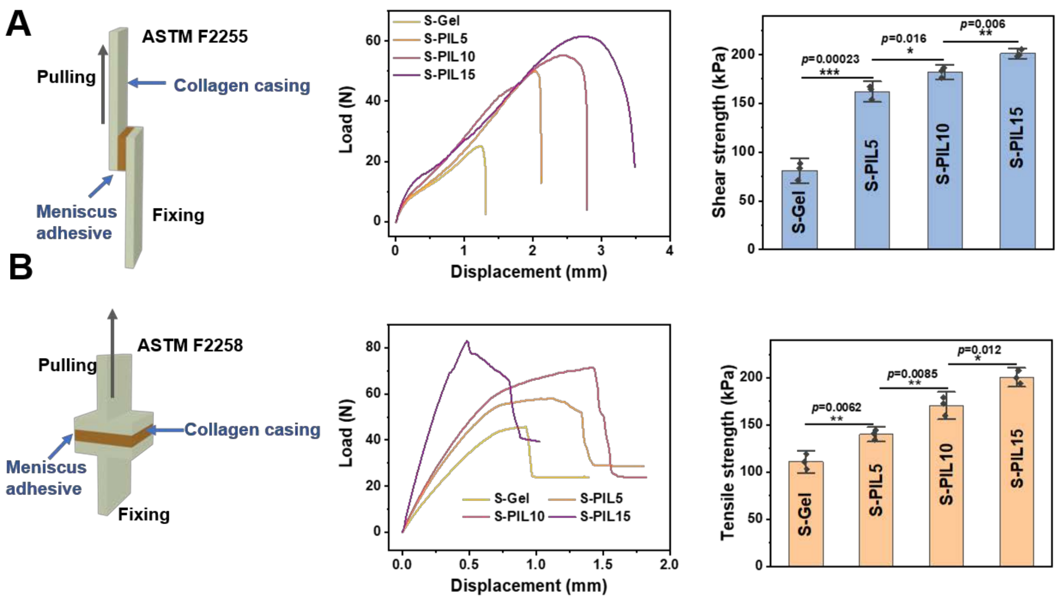

Supplementary Fig. 7 Schematic diagrams with load-displacement curves and shear strength of (A) lap shear testing and (B) tensile testing. Data are presented as mean ± SD (*n* = 3 independent experiments), and exact *p* value was calculated with one-way ANOVA Tukey's multiple comparison test, **p* < 0.05, ***p* < 0.01, ****p* < 0.001, and *****p* < 0.0001.

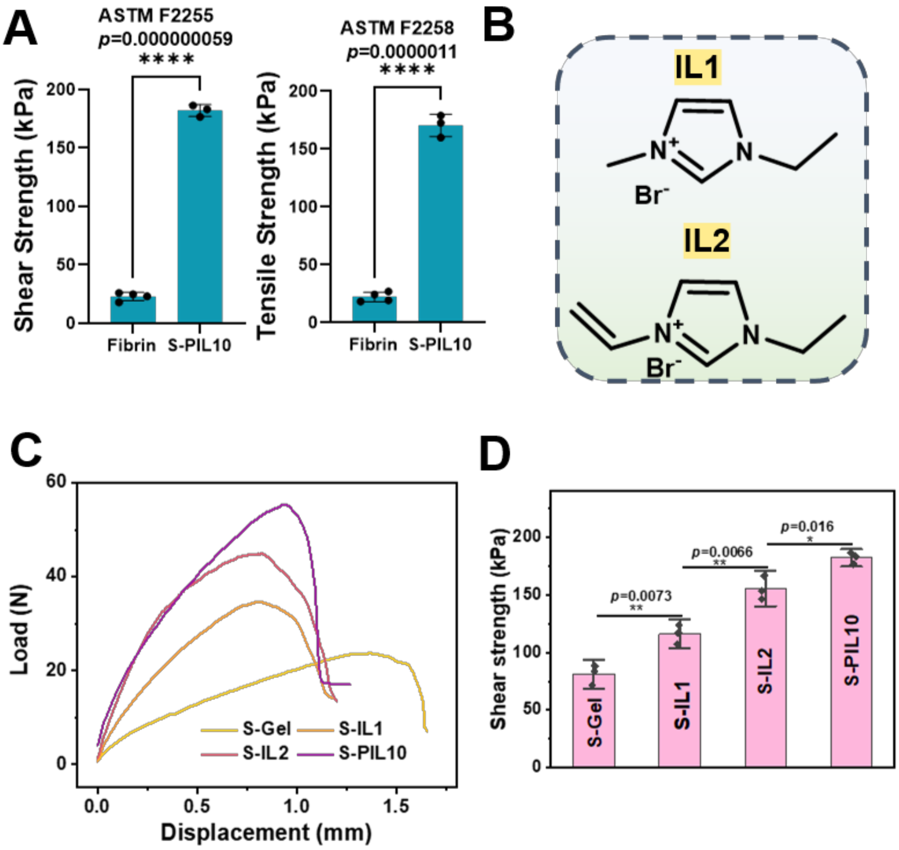

Supplementary Fig. 8 (A) Lap shear strength with fibrin and S-PIL10. Data are presented as mean ± SD (n = 3 independent experiments) and exact p value was calculated with two-tailed student's t-tests. (B) Chemical structure of IL-1 and IL-2. (C) Load-displacement curves of samples with the different ionic liquids. (D) shear strength of different samples. Data are presented as mean ± SD (n = 3 independent experiments), and exact p value was calculated with one-way ANOVA Tukey's multiple comparison test, *p < 0.05, **p < 0.01, ***p < 0.001, and ****p < 0.0001.

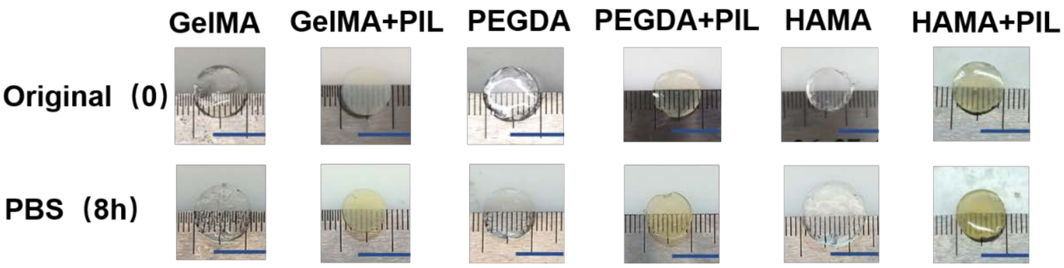

Supplementary Fig. 9 Swelling conditions of GelMA, PEGDA and HAMA with PIL in PBS buffer. Scale bar: 10 mm.

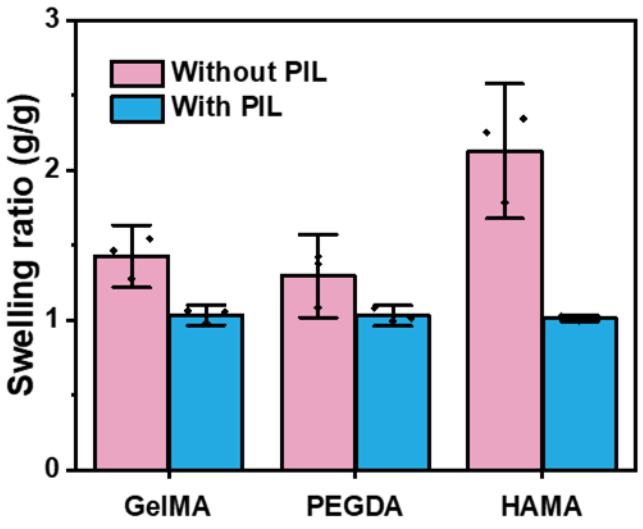

Supplementary Fig. 10 Swelling ratio of GelMA, PEGDA and HAMA with PIL in PBS buffer. Data are presented as mean ± SD (*n* = 3 independent hydrogels).

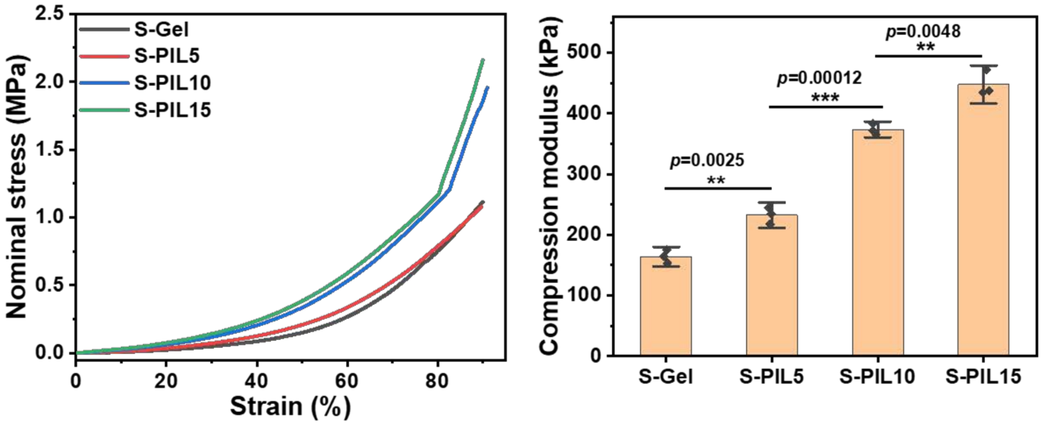

Supplementary Fig. 11 Stress-strain curves of S-Gel with PIL at the different concentrations and compression modulus of S-Gel with the different concentrations of PIL. Data have been presented as mean ± SD, n = 3 independent experiments. Statistically significant differences, as analyzed using one-way ANOVA followed by Tukey's multiple comparison test, have been indicated as *p < 0.05, **p < 0.01, ***p < 0.001, and ****p < 0.0001.

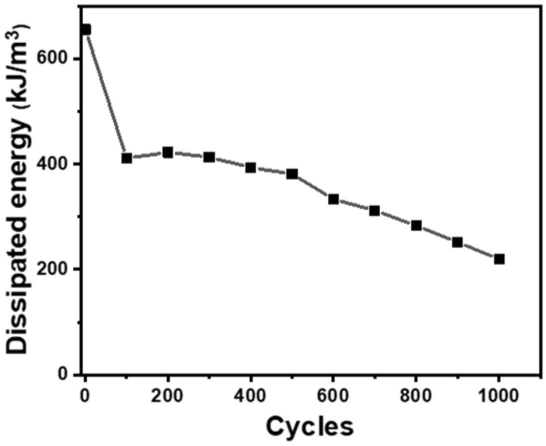

Supplementary Fig. 12 The energy dissipation during loading-unloading tests for 1000 cycles.

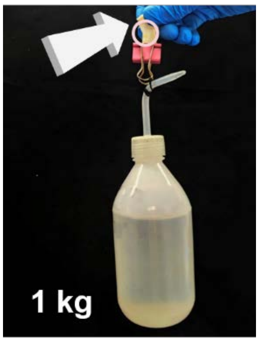

Supplementary Fig. 13 Macroscopic adhesive performance of S-PIL10.

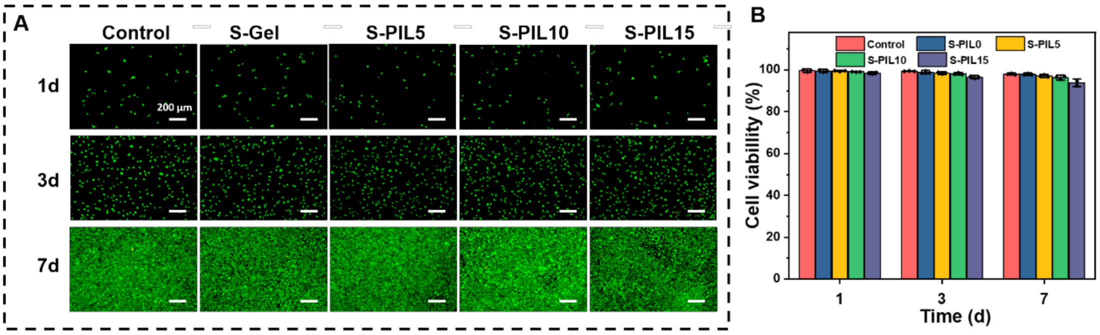

Supplementary Fig. 14 (A) Live/dead cell assays of L929 fibroblasts on the surface of hydrogels (live cells in green and dead cells in red). (B) Cell viability of L929 fibroblasts on the hydrogels within 7 days. Data are presented as mean ± SD (*n* = 3 independent cell experiments). Scale bar: 200 μm.

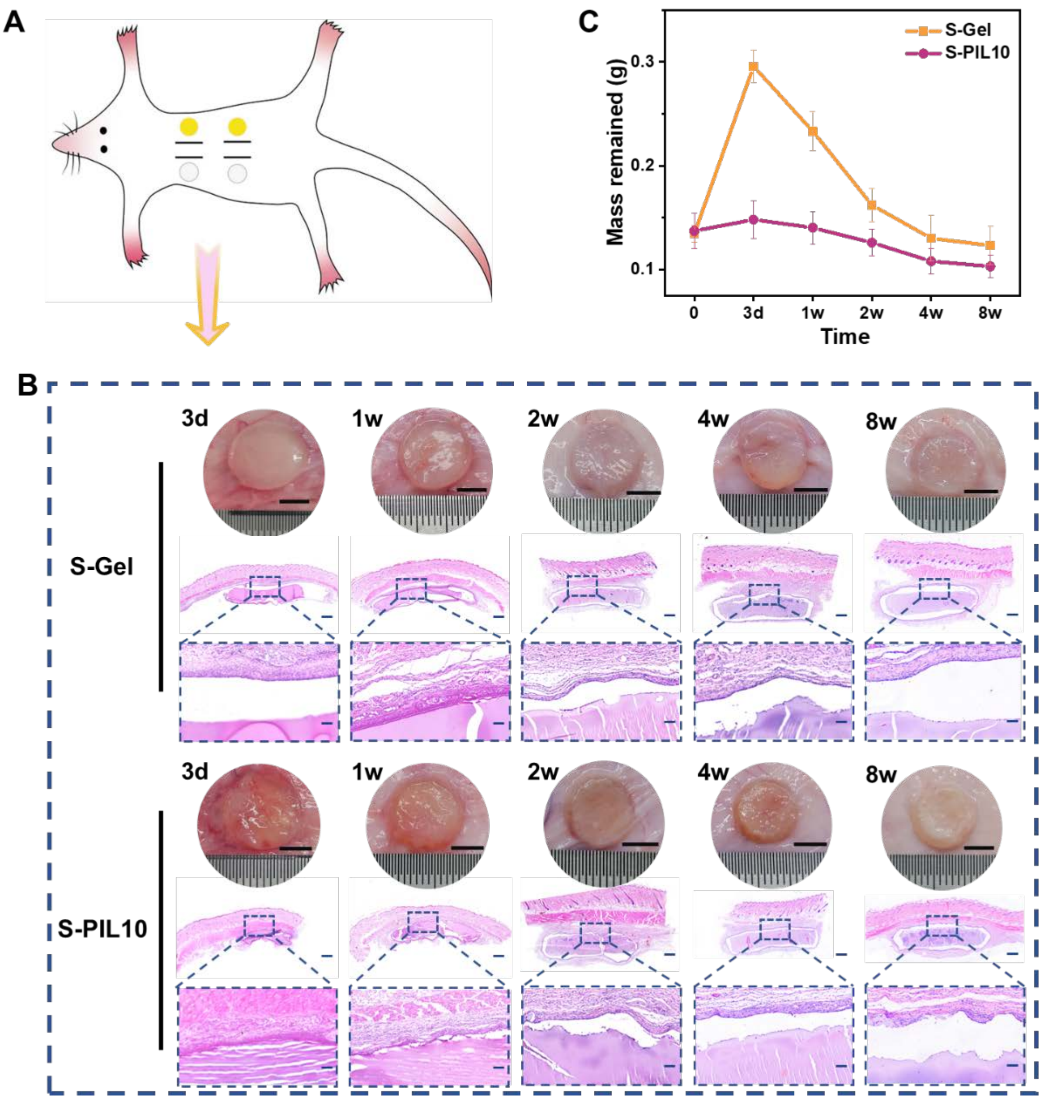

Supplementary Fig. 15 (A) Schematic illustration of Subcutaneous *in vivo* degradation. (B) Macrograph and Hematoxylin & eosin (H&E) staining images of implanted S-Gel and S-PIL10 with the surrounding skin at the different time point. (C) The mass remained of S-Gel and S-PIL10. Data are presented as mean ± SD (*n* = 3 independent samples). (Scale bars at 1 and 4 row are 5 mm, Scale bars at 2 and 5 row are 1 mm, Scale bars at 3 and 6 row are 50 μm).

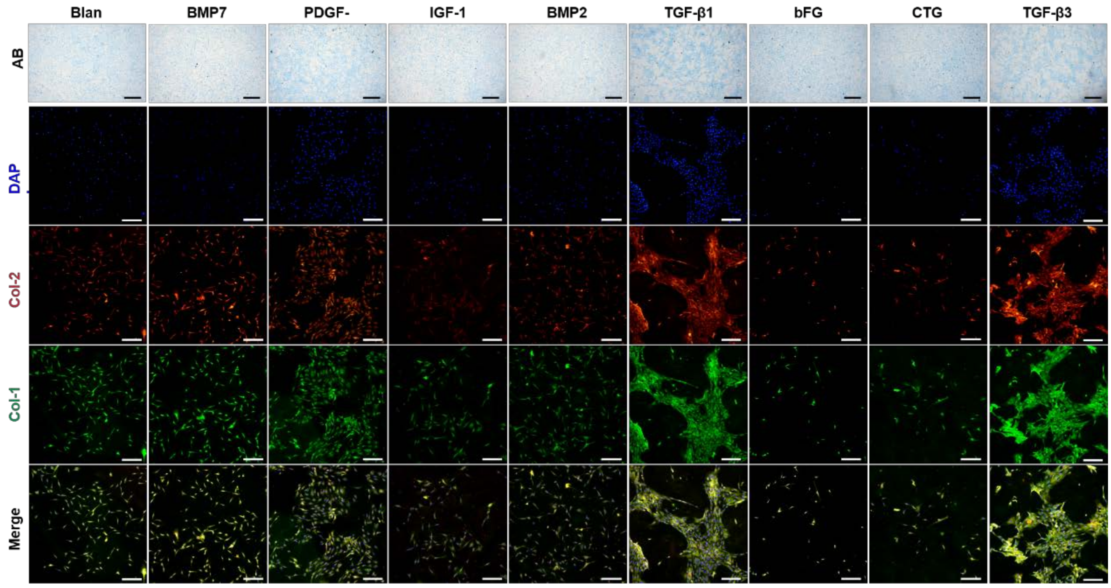

Supplementary Fig. 16 Alcian blue (AB) staining and immunofluorescence staining of meniscus cells treated by the different growth factors. (Scale bar: 200 μm)

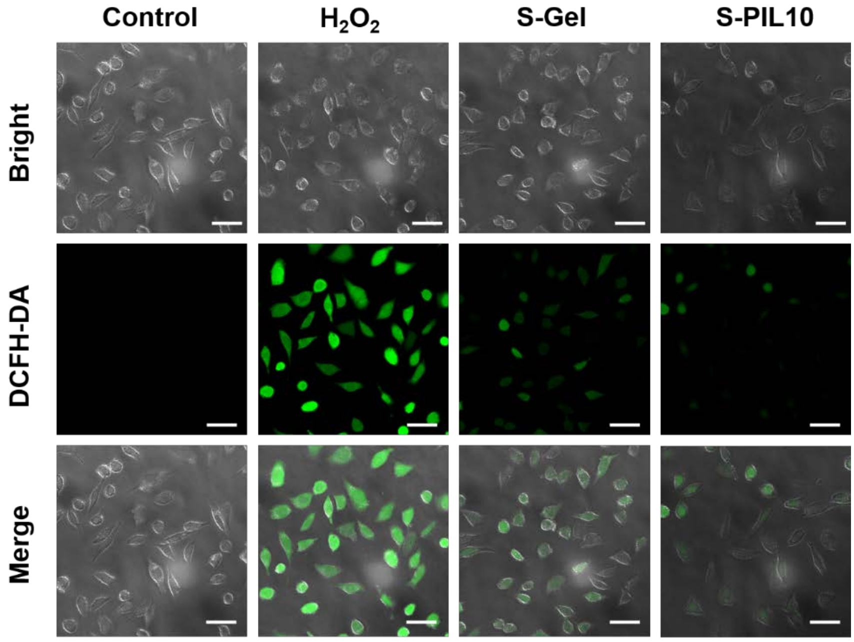

Supplementary Fig. 17 Intercellular ROS scavenging capability of hydrogels evaluated by DCFH-DA (green fluorescence). (Scale bar: 50 μm)

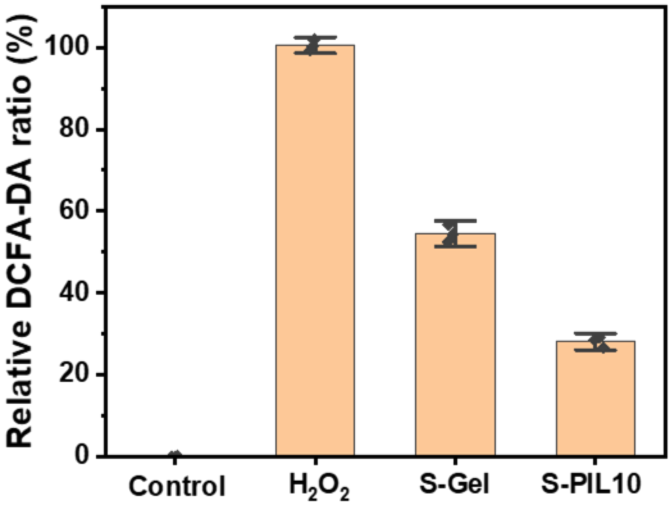

Supplementary Fig. 18 Relative DCFH-DA fluorescence ratio of L929 cells treated with hydrogels. Data are presented as mean ± SD (*n* = 3 independent cell experiments).

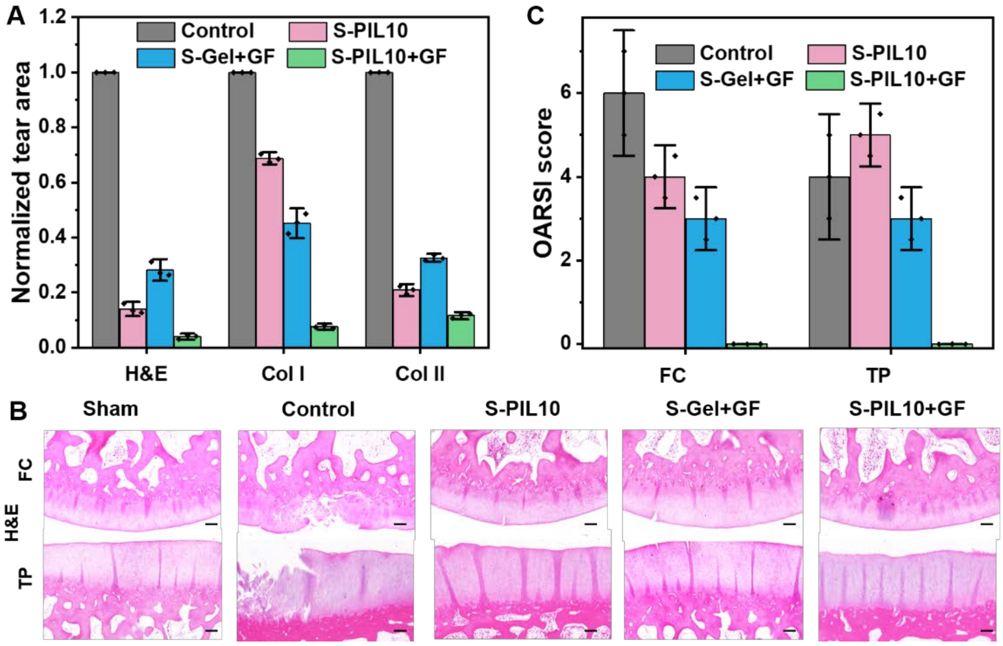

Supplementary Fig. 19 (A) Normalized tear area after meniscus tear repair for two months. Data are presented as mean ± SD (*n* = 3 independent experiments) (B) H&E staining of femoral condyles (FCs) and the tibial plateaus (TPs) after meniscus radial tears. Scale bars are 200 μm. (C) Osteoarthritis Research Society International (OARSI) scores of femoral condyles (FCs) and the tibial plateaus (TPs) after meniscus tear of two months. Data are presented as mean ± SD (*n* = 3 independent experiments).

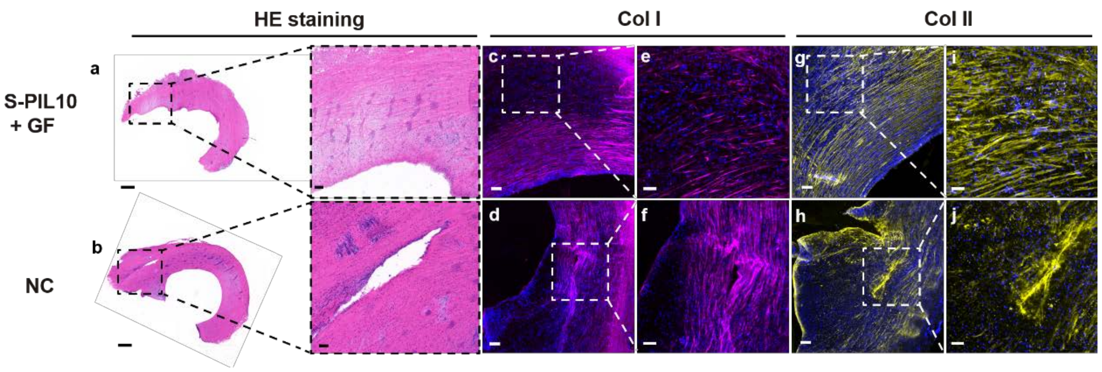

Supplementary Fig. 20 H&E staining and immunofluorescence staining of S-PIL10 with TGF-β1 group and negative control with circumferential tears at 2 months. Scale bars in 1 column are 1 mm, other scale bars are 100μm.

Supplementary Table 1 Composition ratios used to fabricate the meniscus adhesives

Designation / Ingredient	S-Gel	S-PIL5	S-PIL10	S-PIL15
**SFMA (mg/mL)**	300	300	300	300
**LAP (mg/mL)**	2.5	2.5	2.5	2.5
**PIL (mg/mL)**	0	5	10	15

Supplementary Table 2 Element content analysis of samples by XPS survey.

Sample	Percentage (mol %)			
	C1s	O1s	N1s	B1s
**S-Gel**	64.03	20.90	15.07	0
**S-PIL5**	61.69	20.53	16.37	1.41
**S-PIL10**	58.86	21.59	17.61	1.94
**S-PIL15**	57.01	21.79	18.82	2.38
