# Supplementary material for: Silk fibroin hydrogel adhesive enables sealed-tight reconstruction of meniscus tears
Source: Nat Commun. 2024 Mar 26;15:2651. doi: 10.1038/s41467-024-47029-6 (PMC10966011; doi:10.1038/s41467-024-47029-6)
Supplement: Supplementary file 2 — Reporting Summary [file 41467_2024_47029_MOESM2_ESM.pdf]

nature portfolio

Corresponding author(s): Hongwei Ouyang

Last updated by author(s): Feb 23, 2024

Reporting Summary

Nature Portfolio wishes to improve the reproducibility of the work that we publish. This form provides structure for consistency and transparency in reporting. For further information on Nature Portfolio policies, see our Editorial Policies and the Editorial Policy Checklist.

Statistics

For all statistical analyses, confirm that the following items are present in the figure legend, table legend, main text, or Methods section.

n/a	Confirmed	
☐	☒	The exact sample size (*n*) for each experimental group/condition, given as a discrete number and unit of measurement
☐	☒	A statement on whether measurements were taken from distinct samples or whether the same sample was measured repeatedly
☐	☒	The statistical test(s) used AND whether they are one- or two-sided<br>*Only common tests should be described solely by name; describe more complex techniques in the Methods section.*
☒	☐	A description of all covariates tested
☒	☐	A description of any assumptions or corrections, such as tests of normality and adjustment for multiple comparisons
☐	☒	A full description of the statistical parameters including central tendency (e.g. means) or other basic estimates (e.g. regression coefficient) AND variation (e.g. standard deviation) or associated estimates of uncertainty (e.g. confidence intervals)
☐	☒	For null hypothesis testing, the test statistic (e.g. *F*, *t*, *r*) with confidence intervals, effect sizes, degrees of freedom and *P* value noted<br>*Give P values as exact values whenever suitable.*
☒	☐	For Bayesian analysis, information on the choice of priors and Markov chain Monte Carlo settings
☒	☐	For hierarchical and complex designs, identification of the appropriate level for tests and full reporting of outcomes
☒	☐	Estimates of effect sizes (e.g. Cohen's *d*, Pearson's *r*), indicating how they were calculated

Our web collection on statistics for biologists contains articles on many of the points above.

Software and code

Policy information about availability of computer code

Data collection	Chemical compositions were analyzed by the X-ray photoelectron spectroscopy (XPS, K-Alpha, Thermo Fisher Scientific, USA) and the Fourier transform infrared spectroscopy (FTIR, Vertex 70, Bruker, Germany).<br>The cross-sectional morphology of the samples was observed through SEM (EM-30+, COXEM, South Korea). The proton signals of the sample was collected by nuclear magnetic resonance (NMR, AVANCE NEO 400, Bruker, Germany).<br>The mechanical properties of the samples were tested via a mechanical tester (Instron 5943, Instron, USA).<br>Extracellular antioxidant results were obtained by NanoDrop instrument (Thermo Fisher Scientific, USA).<br>Live death assay and immunofluorescence images was recored by inverted microscope (Nikon-LV150N, Nikon, Japan), and histological images were collected by Pannoramic MIDI scanner (3D HISTECH, Hungary).
Data analysis	Origin 2023 software and GraphPad Prism9.0. were used for data plotting and statistical analysis.<br>Image J software was used for quantitative imaging analyses.<br>XPSPEAK software was used for XPS analysis. PeakFit v4.12 software was used to analyze the secondary structure of protein samples.

For manuscripts utilizing custom algorithms or software that are central to the research but not yet described in published literature, software must be made available to editors and reviewers. We strongly encourage code deposition in a community repository (e.g. GitHub). See the Nature Portfolio guidelines for submitting code & software for further information.

Data

Policy information about availability of data

All manuscripts must include a data availability statement. This statement should provide the following information, where applicable:

- Accession codes, unique identifiers, or web links for publicly available datasets
- A description of any restrictions on data availability
- For clinical datasets or third party data, please ensure that the statement adheres to our policy

All the data supporting the findings of this study are available within the main text of this article and its Supplementary Information. Any additional requests for information can be directed to, and will be fulfilled by the corresponding authors. Source data are provided with this paper.

Research involving human participants, their data, or biological material

Policy information about studies with human participants or human data. See also policy information about sex, gender (identity/presentation), and sexual orientation and race, ethnicity and racism.

Reporting on sex and gender	This work did not involve human research.
Reporting on race, ethnicity, or other socially relevant groupings	This work did not involve human research.
Population characteristics	This work did not involve human research
Recruitment	This work did not involve human research
Ethics oversight	This work did not involve human research

Note that full information on the approval of the study protocol must also be provided in the manuscript.

Field-specific reporting

Please select the one below that is the best fit for your research. If you are not sure, read the appropriate sections before making your selection.

☒ Life sciences ☐ Behavioural & social sciences ☐ Ecological, evolutionary & environmental sciences

For a reference copy of the document with all sections, see nature.com/documents/nr-reporting-summary-flat.pdf

Life sciences study design

All studies must disclose on these points even when the disclosure is negative.

Sample size	Sample sizes were chosen based on state-of-the-art work available in the literature investigating tissue adhesion ex vivo. Please refer to:<br>1. Anthis, Alexandre HC, et al. "Modular stimuli-responsive hydrogel sealants for early gastrointestinal leak detection and containment." Nature Communications 13.1 (2022): 7311.<br>2. Yang, Yuxuan, et al. "Ultra-durable cell-free bioactive hydrogel with fast shape memory and on-demand drug release for cartilage regeneration." Nature Communications 14.1 (2023): 7771.
Data exclusions	No data were excluded from this study.
Replication	All experiments were performed with independent replicates as described in the figure legends.
Randomization	All samples were randomly allocated into experimental groups.
Blinding	Investigators were blinded to group allocation during data collection and analysis.

Reporting for specific materials, systems and methods

We require information from authors about some types of materials, experimental systems and methods used in many studies. Here, indicate whether each material, system or method listed is relevant to your study. If you are not sure if a list item applies to your research, read the appropriate section before selecting a response.

Materials & experimental systems

n/a	Involved in the study	
	x	Antibodies
	x	Eukaryotic cell lines
x		Palaeontology and archaeology
	x	Animals and other organisms
x		Clinical data
x		Dual use research of concern
x		Plants

Methods

n/a	Involved in the study	
x		ChIP-seq
x		Flow cytometry
x		MRI-based neuroimaging

Antibodies

Antibodies used	Antibodies used For immunofluorescence staining, cryo-sectioned samples were incubated with Anti-Collagen ll antibody (diluted 1:100, NB600-844, NOVUS, USA) and Anti-Collagen l antibody (diluted 1:200, ab88147, Abcam, USA). Secondary antibodies used are as follows: Goat anti-mouse IgG H&L (Alexa Fluor® 555, ab150114, Abcam, USA) and Goat Anti-Mouse IgG H&L (Alexa Fluor® 488, ab150113 Abcam, USA).
Validation	Validation details of the primary antibodies are available on the manufacturers' websites:NB600-844 (https://www.novusbio.com/products/collagen-ii-antibody-5b25_nb600-844) ab88147 (https://www.abcam.cn/products/primary-antibodies/collagen-i-antibody-3g3-bsa-and-azide-free-ab88147.html)

Eukaryotic cell lines

Policy information about cell lines and Sex and Gender in Research

Cell line source(s)	L929 cell lines were purchased from iCell Bioscience (Shanghai, China) and derived from mice. The rabbit meniscus cells were extracted from the 16-week-old New Zealand white rabbit and cultured with F12 medium. The cell line sources did not take gender into account.
Authentication	L929 cell lines were authenticated by the supplied using Short Tandem Repeat test. The rabbit meniscus cells haven't been authenticated.
Mycoplasma contamination	These cell lines were not tested for mycoplasma contamination.
Commonly misidentified lines (See ICLAC register)	None

Animals and other research organisms

Policy information about studies involving animals; ARRIVE guidelines recommended for reporting animal research, and Sex and Gender in Research

Laboratory animals	Male Sprague-Dawley rats (SD rats, 8 weeks old) and Newzealand white Rabbits (16 weeks old) were used for all our experiments.
Wild animals	This study did not involve wild animals.
Reporting on sex	This study did not apply to only one sex. The study design and methods did not take sex into consideration. All male animals were used in this study, and sex differences were not considered. Animal experiments were not considered for sex analysis because meniscus structure and functions in the animal model and inflammatory responses in subcutaneous encapsulation were not related to sex.
Field-collected samples	This study did not involve samples collected from the field.
Ethics oversight	All animal experiments have been approved by the Animal Experiment Committee of Zhejiang University and fully comply with the National Institutes of Health Guide for the Care and Use of Laboratory Animals (AIRB-2022-0483).

Note that full information on the approval of the study protocol must also be provided in the manuscript.

Plants

Seed stocks	This study did not involved plants.
Novel plant genotypes	This study did not involved plants.
Authentication	This study did not involved plants.
