# Supplementary material for: Silk fibroin hydrogel adhesive enables sealed-tight reconstruction of meniscus tears
Source: Nat Commun. 2024 Mar 26;15:2651. doi: 10.1038/s41467-024-47029-6 (PMC10966011; doi:10.1038/s41467-024-47029-6)
Supplement: Supplementary file 3 — Description of Additional Supplementary Files [file 41467_2024_47029_MOESM3_ESM.pdf]

Supplementary Movie 1
The performance of shear adhesion of the meniscus adhesive. The adhered glass slide adhere through adhesive lifted firmly a bucket (＞5 kg).

Supplementary Movie 2
The adhesion performance of the adhesive to the meniscus. A metal grinding pestle with appropriate size was used to simulate the pressure of the femur on the meniscus, and the adhered meniscus showed no significant changes.
